# Editorial: Plants in karst degraded habitats: diversity, evolution, and ecological functions

**DOI:** 10.3389/fpls.2026.1997887

**Published:** 2026-09-29

**Authors:** Hao Zhang, Yili Guo, Hu Du, Akram Salah

**Affiliations:** 1 Huanjiang Observation and Research Station for Karst Ecosystem, Guangxi Key Laboratory of Karst Ecological Processes and Services, Institute of Subtropical Agriculture, Chinese Academy of Sciences, Changsha, China; 2 College of Environment and Resource, Guangxi Normal University, Guilin, China; 3 Lasbela University of Agriculture, Water and Marine Sciences, Uthal, Pakistan

**Keywords:** adaptive evolution, ecosystem multifunctionality, functional traits, karst plants, plant diversity

Karst landscapes cover approximately 22 million km^2^ of Earth’s surface, of which about 5.1 million km^2^ is exposed carbonate rock concentrated in the East Asian, Mediterranean, and eastern North American karst regions (Guo et al., 2026). These landscapes rank among the most heterogeneous and stressful terrestrial environments: thin, discontinuous soils, high rock exposure, intense seasonal drought, and elevated calcium availability impose severe filters on plant life. In response, karst floras have evolved distinctive syndromes—lithophyly, xerophyly, and calcium tolerance—and harbor exceptionally high biodiversity, including numerous endemic and endangered species (Sun et al., 2026a). Yet karst ecosystems are also among the most threatened: in southwestern China alone, rocky desertification has degraded vast areas of the largest continuous karst terrain on Earth (~540,000 km^2^), with profound consequences for soil and water conservation, biodiversity, and livelihoods (Zhang et al., 2025). Understanding how plants assemble, adapt, and function in these degraded habitats is therefore both a fundamental ecological question and an urgent management imperative. This Research Topic was conceived to advance that understanding across three intertwined axes—diversity, evolution, and ecological function—by integrating community ecology, functional trait biology, and restoration science.

A first cluster of contributions clarifies how environmental filtering and dispersal jointly structure karst plant communities. Working in a subtropical karst forest, Long et al. demonstrate that environmental filtering and dispersal limitation together shape taxonomic, functional, and phylogenetic diversity, underscoring that niche and neutral processes operate concurrently. Complementing this, Sun et al. show that habitat filtering dominates the assembly of primary forest in a karst degradation region, with topography and soil outweighing phylogenetic history in driving functional trait variation. Across successional stages, Wang et al. reveal that species and functional diversity respond increasingly strongly to soil factors—particularly the N:P ratio, C:N ratio, bulk density, phosphorus, and organic matter—as succession proceeds. At the landscape scale, Zhang et al. identify a nutrient advantage in peak-cluster depressions that structures plant diversity responses, highlighting fine-grained habitat heterogeneity as a key organizing force.

A second set of papers illuminates the adaptive trait strategies that permit plant persistence on karst. Yao et al. distinguish the drought-resistance strategies and indicator traits of *Platycladus orientalis* and *Broussonetia papyrifera*, while He et al. show that soil filtering of *Pinus yunnanensis* leaf functional traits diverges between karst and non-karst habitats, shifting from pH screening toward stoichiometric control. Extending trait-based inquiry to the often-ignored cryptogamic component, Hu et al. document functional trait variation and adaptive strategies of epilithic bryophytes in degraded karst ecosystems. Together these studies refine our mechanistic understanding of how trait–environment matching generates the characteristic karst flora, while reminding us that non-vascular and whole-plant perspectives remain essential.

A third theme concerns ecosystem functioning and restoration. Chu et al. demonstrate that vegetation restoration enhances soil aggregate stability and aggregate-associated black carbon in a rocky desertification area of southwestern Guangxi, directly linking the recovery of plant cover to soil structure and carbon sequestration. Qi et al. show that plant functional diversity promotes ecosystem multifunctionality in abandoned karst farmland and, critically, that this relationship is modulated by changing rainfall patterns—a finding with clear implications under climate change. In a complementary analysis, Zheng et al. show that soil fertility context shapes biodiversity–carbon associations across forest strata. These results collectively reinforce the insurance hypothesis: maintaining functional diversity is central to sustaining multiple ecosystem services in fragile karst landscapes.

Finally, two studies broaden the functional lens to biotic interactions and stand dynamics. Wang et al. resolve the seasonal dynamics and functional diversity of frugivorous bird–plant interaction networks, revealing how animal-mediated processes structure karst communities. Li et al. show that stand spatial structure promotes tree growth and sapling diversity in northern tropical karst seasonal rainforest, pointing to structural complexity as a lever for management.

Collectively, these twelve studies substantially advance the field, yet important gaps remain. First, as noted in our original call, genetic diversity is still underrepresented relative to species and functional diversity; linking genomic and quantitative genetic approaches to field ecology promises to reveal the evolutionary potential of karst lineages. Second, the belowground realm—root functional ecology, plant–soil microbe feedbacks, and multi-trophic cascades—remains technically challenging to study in fractured karst substrates and demands methodological innovation, such as minirhizotron imaging, isotope tracing, and metabolomics (Zhang et al., 2025; Sun et al., 2026b). Third, most contributions derive from the East Asian karst region; comparative work across the Mediterranean rim and eastern North American karsts is needed to test the generality of assembly and adaptation rules. Fourth, long-term observations and manipulative experiments, particularly factorial rainfall and nutrient manipulations, are required to move from pattern to process and to predict responses to climate change (Lu et al., 2026). Finally, greater attention to endangered and economically important karst plants will strengthen the bridge between conservation science and practice, as will the integration of trait-based indicators into rocky-desertification control and ecological restoration programs.

Karst degraded habitats are natural laboratories for testing ecological theory under stress, and the studies assembled here show that progress comes from combining taxonomic, functional, and phylogenetic perspectives with soil, climatic, and biotic contexts. We thank the contributing authors, reviewers, and the editorial team at *Frontiers in Plant Science*, and we hope this collection stimulates the interdisciplinary, methodologically innovative, and globally comparative research that karst ecosystems urgently need.
